# Visualization and Identification of IL-7 Producing Cells in Reporter Mice

**DOI:** 10.1371/journal.pone.0007637

**Published:** 2009-11-10

**Authors:** Renata I. Mazzucchelli, Søren Warming, Scott M. Lawrence, Masaru Ishii, Mehrnoosh Abshari, A. Valance Washington, Lionel Feigenbaum, Andrew C. Warner, Davis J. Sims, Wen Qing Li, Julie A. Hixon, Daniel H. D. Gray, Benjamin E. Rich, Matthew Morrow, Miriam R. Anver, James Cherry, Dieter Naf, Lawrence R. Sternberg, Daniel W. McVicar, Andrew G. Farr, Ronald N. Germain, Keith Rogers, Nancy A. Jenkins, Neal G. Copeland, Scott K. Durum

**Affiliations:** 1 Laboratory of Molecular Immunoregulation, Cancer and Inflammation Program, Center for Cancer Research, National Cancer Institute, National Institute of Health, Frederick, Maryland, United States of America; 2 Mouse Cancer Genetics Program, National Cancer Institute, Frederick, Maryland, United States of America; 3 Laboratory Animal Science Program (LASP), Science Applications International Corporation (SAIC), Cancer Research Center, National Cancer Institute, Frederick, Maryland, United States of America; 4 Lymphocyte Biology Section, Laboratory of Immunology, National Institute of Allergy and Infectious Diseases, National Institutes of Health, Bethesda, Maryland, United States of America; 5 Laboratory of Experimental Immunology, Cancer and Inflammation Program, Center for Cancer Research, National Cancer Institute, National Institute of Health, Frederick, Maryland, United States of America; 6 The Walter and Eliza Hall Institute of Medical Research, Parkville, Victoria, Australia; 7 Department of Dermatology, Brigham and Women's Hospital, Boston, Massachusetts, United States of America; 8 Human Retrovirus Section, Vaccine Branch, Center for Cancer Research NCI, Frederick, Maryland, United States of America; 9 Gene Expression Laboratory, SAIC-Frederick, Inc., NCI-Frederick, Frederick, Maryland, United States of America; 10 Department of Immunology, University of Washington, Seattle, Washington, United States of America; New York University School of Medicine, United States of America

## Abstract

Interleukin-7 (IL-7) is required for lymphocyte development and homeostasis although the actual sites of IL-7 production have never been clearly identified. We produced a bacterial artificial chromosome (BAC) transgenic mouse expressing ECFP in the *Il7* locus. The construct lacked a signal peptide and ECFP (enhanced cyan fluorescent protein ) accumulated inside IL-7-producing stromal cells in thoracic thymus, cervical thymus and bone marrow. In thymus, an extensive reticular network of IL-7-containing processes extended from cortical and medullary epithelial cells, closely contacting thymocytes. Central memory CD8 T cells, which require IL-7 and home to bone marrow, physically associated with IL-7-producing cells as we demonstrate by intravital imaging.

## Introduction

Interleukin-7 (IL-7) is required for T cell development and survival (reviewed in Khaled and Durum [1]) as first appreciated from the severe lymphopenia observed in IL7^−/−^ and IL-7R^−/−^ mice [2], [3], then in comparable deficiencies in humans found to lack components of the IL-7 receptor [4] (reviewed in Puel and Leonard [5]).

Although IL-7 plays a critical role in the thymus and peripheral T cell homeostasis, stromal cells producing IL-7 have never been precisely identified. This was mainly due to the low abundance of message and protein (as we demonstrate in this study). Since IL-7 was first described in 1988, a number of studies have detected *Il7* message in various tissues using Northern blot analysis or RT-PCR. These *Il7* mRNA-expressing tissues include human and mouse thymus and spleen [6], [7], mouse kidney [6], mouse fetal thymus [8]–[11], mouse fetal intestine and liver [11], [12] and adult human liver [13]. In our own lab, we have verified the presence of *Il7* mRNA by RT-PCR from homogenized mouse tissue including thymus, spleen, lymph nodes and bone marrow (R.I. Mazzucchelli, unpublished observations). RT-PCR has also been used to identify *Il7* mRNA in specific cell populations including those derived from human tonsillar germinal centers [14], fetal thymus stromal cells [15], mouse bone marrow stromal cells [16], mouse and human keratinocytes [17]–[20], human intestinal epithelial cells [21], [22], human follicular dendritic cells and vascular cells [23], human mature peripheral dendritic cells [14], [24] and human platelets [25].

There are a few reports identifying sites of *Il7* mRNA production using in situ hybridization which indicate transcription in human postnatal thymus [26] and mouse embryonic, postnatal and adult thymus [27], [28], mouse and human keratinocytes [17] and human intestinal mucosa [21]. In mouse thymus, *Il7* mRNA expression was reported in one study to decline in adulthood to below the level of detection by in situ hybridization [28]. In another study [27], it was reported that the adult thymus sections required 6 weeks of exposure to the probe to develop a clear signal. Yet despite IL-7 being virtually undetectably by in situ hybridization in the adult, it is clear that the adult mouse thymus produces biologically significant IL-7 based on thymic reconstitution experiments that show a dramatic difference between IL-7^−/−^ compared to wild type thymus.

The production of *Il7* mRNA does not guarantee that a cell produces the protein because posttranscriptional controls can block mRNA translation. This is the case for IL-15, a cytokine related to IL-7 and with similar homeostatic activities. Production of IL-15 is regulated not only by transcription and mRNA stability, like most cytokines, but also at the translation level (reviewed by Tagaya et al. [29] The 5′ untranslated region of *Il15* mRNA contains 10 ATG sequences which strongly inhibit translation. Similarly, the 5′ untranslated region of murine *Il7* mRNA contains 8 ATG sequences and has also been shown to greatly inhibit translation in Cos-7 cells [6]. In our laboratory, we analyzed 20 stromal cell lines from mouse thymus, bone marrow and spleen, all expressing *Il7* mRNA, but only two produced enough protein to be detectable by ELISA or bioassay (R.I. Mazzucchelli, unpublished observations) suggesting that there could be translational inhibition of IL-7 production.

Immunohistochemical detection of IL-7 protein in human tissue has been reported by several groups. Although not reported in human thymus, immunohistochemical reactions have been seen in healthy human intestinal epithelial cells [21], human follicular dendritic cells[23], human *Schistosoma mansoni* infected skin [20], human Warthin's tumor [30], healthy human liver [13] and lymph nodes of AIDS patients [31]. The specificity of such staining can be more easily assessed using mouse tissues because of the availability of IL-7^−/−^ mice. There are some early reports of positive immunohistochemical reactions for murine IL-7 in adult bone marrow [32], fetal liver tissue [33], embryonic [9], [34] and adult thymus [33], all preceding the availability of IL-7^−/−^ tissues to verify specificity. An experienced veterinary pathology laboratory at NCI tested six different commercial and non-commercial anti-IL-7 monoclonal and polyclonal antibodies, following published protocols and optimizing our own protocols. No specific reactions were found in any mouse lymphoid or non-lymphoid tissues, some of those data are shown in the results.

Because the identification of IL-7-producing cells has been so difficult, we have developed a novel strategy to amplify the signal from these cells and allow direct visualization of such cells in tissues. A bacterial artificial chromosome was modified to introduce a fluorescent reporter gene into the *Il7* locus that should be trapped inside the producing cells. Transgenic mice prepared using this reporter construct enabled, for the first time, identification of the cells that produce IL-7 in thymus and bone marrow.

## Results

### IL-7 expression in thymus is below the level of detection by immunohistochemistry and in situ hybridization

We and others (personal communications: D. Klug, NCI; C. Mackall, NCI; C. Willis, Amgen) have tested a number of polyclonal and monoclonal anti-IL-7 antibodies and failed to observe a positive reaction in thymic tissue (Table 1). Some of these antibodies are effective in western blotting or blocking the biological activity of IL-7. Immunohistochemical methods, used extensively in our institute, were applied to thymic tissue from C57BL/6, Rag2^−/−^, and as a positive control, mice overexpressing IL-7 under control of the *K14* promoter. Thymus tissue from IL-7^−/−^ mice was used as a negative control. Several protocols for tissue preparation were evaluated that showed staining with the positive control (transgenic overexpression of IL-7), but all were consistently negative with normal thymus, except two that apparently gave non-specific staining based on a signal from IL-7^−/−^ thymic material.

**Table 1:** Immunohistochemistry fails to detect IL-7 in murine thymus.

	Antibody	Notes	Results
1	Rabbit Polyclonal IgG^1^	Epitope mapping at the N-terminus	Negative
2	Goat Polyclonal IgG^2^	Epitope mapping at the C-terminus	Negative
3	Goat Polyclonal IgG^3^	Purified by affinity chromatography	Negative
4	Monoclonal Rat IgG^4^	Purified by affinity chromatography	Negative
5	Rabbit Polyclonal IgG^5^	Purified by affinity chromatography and biotinylated	Negative
6	Monoclonal Mouse IgG^6^	Effective blocking in vivo and in vitro	Negative

Several commercial and non-commercial anti-IL-7 antibodies were tested at various concentration and fixation conditions and evaluated by a veterinary pathologist. None of them showed positive staining of normal thymus although several reacted non-specifically with an IL7^−/−^ mouse tissue. Sources: 1–Santa Cruz, 2–Santa Cruz, 3–R&D, 4–R&D, 5–Peprotech, 6–Amgen.

Because mRNA could be more readily detectable than protein if there were impediments to translation in case IL-7 synthesis were subjected to translational or post-translational control, we examined expression of *Il7* mRNA in the thymus by in situ hybridization. Three different set of probes were synthesized and tested on both frozen or paraffin embedded tissue. The same mouse strains (C57BL/6, Rag2^−/−^ and K14) that failed to show detectable IL-7 by immunohistochemistry also failed to give a signal for *Il7* mRNA by in situ hybridization (data not shown).

**Figure 1:**
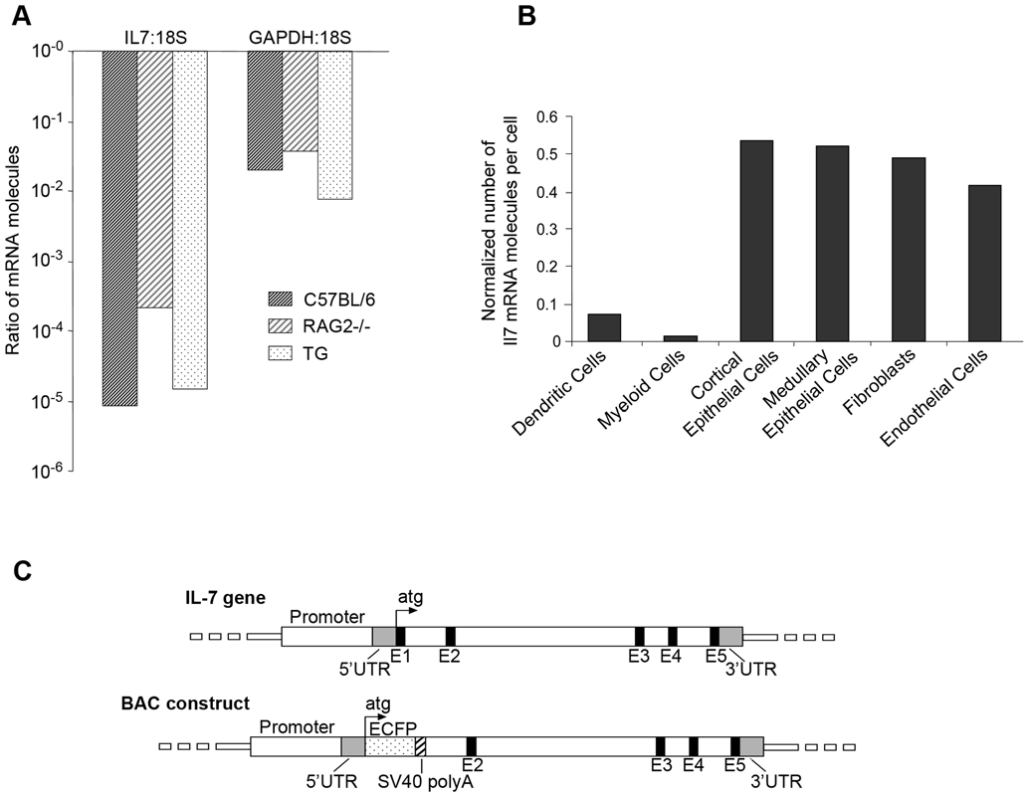
Il7 mRNA expression in different mouse strains and in thymic stromal cell subtypes. A. Thymi from C57BL/6, Rag2^−/−^ and transgenic (TG) mice expressing IL-7 under control of the K14 promoter were analyzed for levels of *Il7* and *Gapdh* expression normalized to *18 s*. Wild type and even the transgenic mice expressed very low relative levels of *Il7* mRNA, while the Rag2^−/−^ showed somewhat more, presumably because the thymus is enriched for stromal cells. *Gapdh* was expressed at a comparable level among all the strains. Data are the mean of three mice, no standard deviations are shown because ratios were used in the calculations. B. Quantification of *Il7* mRNA molecules in thymic stromal cells. Stromal cell subsets were sorted using specific surface markers (Table 1B). RNA was extracted and absolute real time PCR quantification was performed. *Il7* mRNA expression was normalized to *Hprt* mRNA expression for each subset. The data shown is representative of at least 2 separations for each subset. C. BAC construct for the IL7promECFP transgenic mouse. The wild type murine *Il7* gene is represented in the upper part of the figure. A BAC containing the mouse *Il7* gene (lower part of the figure) was modified and used to create the transgenic mouse. The exon 1 sequence, encoding the signal peptide was replaced with the *Ecfp* cDNA sequence starting after the ATG start site. The production of ECFP would thus be driven by the *Il7* promoter in IL-7 producing cells. Elimination of the signal peptide was intended to cause ECFP to accumulate inside IL-7 producing cells, enhancing visualization.

Because two routine laboratory techniques, immunohistochemistry and in situ hybridization, failed to localize IL-7 production in thymus, we quantified *Il7* mRNA expression in thymus by real time PCR. Total mRNA was extracted from thymi harvested from C57BL/6, Rag2^−/−^ and K14 mice and immediately placed into RNAlater to protect the mRNA from degradation. Following RNA extraction, RT-real time PCR was performed to compare the expression of *Il7* mRNA to the housekeeping genes *Gapdh* and *18 s*. Data in Figure 1A show that mRNA expression of *Il7* in the whole thymus was 1–2 orders of magnitude lower than *Gapdh* and 4–5 orders of magnitude lower than *18 s* mRNA. This extremely low level of *Il7* mRNA expression in thymus could account for the failure to detect it by in situ hybridization, and in turn the failure to detect the protein by immunohistochemistry, although other technical explanations could also account for it, if for example none of the six anti-IL-7 antibodies are suitable for immunohistochemistry.

### Cell sorting of mouse stromal cell subsets

Because visualization of IL-7 producing cells in thymus was not achievable by immunohistochemistry or in situ hybridization we aimed to physically purify subsets of thymic stromal cells and quantitate their content of *Il7* mRNA. Table 2 lists the stromal cell subsets known to be present in thymus (dendritic cells, myeloid-derived cells, cortical epithelial cells, medullar epithelial cells, fibroblasts and endothelial cells) and the antibodies used to isolate them. After cell sorting, mRNA was extracted, reverse transcribed and an absolute quantification for *Il7* expression was performed by real time PCR. The amount of *Il7* mRNA was very low (Figure 1B), fewer than one molecule per cell in cortical and medullar epithelial cells, whereas fibroblasts and endothelial cells expressed about half a molecule per cell, and dendritic and myeloid cells showed virtually no signal. Because several thymic stromal subsets expressed *Il7* mRNA, this approach did not reveal a single likely source of IL-7 protein; moreover, IL-7 could be regulated translationally, like the related cytokine IL-15 [29]. For these reasons, the production of *Il7* mRNA by a cell type would not prove that the cell made the protein.

**Table 2:** Specific cell markers for thymic stromal cell subsets.

Stromal Cell Subset	CD45.2	CD11c	EpCAM	Ly51	MTS-15	CD31
			G8.8a	CDR1		MTS-12
Dendritic Cells	+	high				
Myeloid-derived Cells	+	Low/−				
Cortical Epithelial Cells	−		+	+		
Medullary Epithelial Cells	−		+	−		
Fibroblasts	−		−		+	
Endothelial Cells	−		−		+/−	+

These markers were chosen to separate each specific stromal cell subtype by cell sorting (adapted from Gray et al. [48]). The same markers were also used in immunohistochemistry as shown in Figure 4.

### Visualization of IL-7 expression by IL7promECFP BAC transgenic mouse

To visualize IL-7 producing cells, we created a BAC transgenic mouse expressing a cyan fluorescent protein (ECFP) under the control of the *Il7* promoter and any other cis regulatory elements contained in a BAC. Using recombineering techniques, we inserted the *ECFP* cDNA sequence immediately after the ATG start site of *Il7*, replacing exon 1 (Figure 1C and S1). The large 5′ untranslated region was retained since this had been shown to inhibit translation of *Il7*, and we aimed to perturb natural regulation as little as possible. Since exon 1 encodes part of the signal peptide for IL-7, elimination of this sequence should cause ECFP to accumulate inside producing cells and enhance visualization. In addition, the splice donor GT sequence from intron 1 was deleted to prevent interference from splicing. Three founder lines of mice were produced and all showed similar expression patterns as will be discussed. The fluorescence emission of ECFP from tissues was relatively weak using the setup on our fluorescent microscope (although it was very bright in the two photon microscope to be discussed later). The signal was enhanced using an antibody against ECFP followed by a fluorescent secondary antibody to generate the images shown–no signal was detected in control tissues from non-transgenic mice.

**Figure 2:**
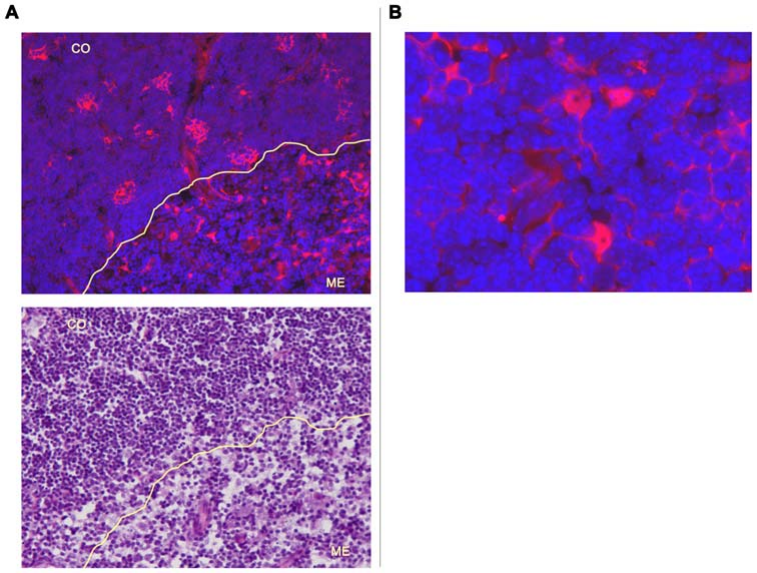
IL-7 is expressed in thymus. A. Expression of ECFP (or IL-7) by stromal cells in thymus is indicated by red fluorescence in the upper panel. The lower panel shows thymocytes by H&E staining on a sequential slide. The lines separate cortex (CO) from medulla (ME) in both pictures. The expression of IL-7 is higher in cortex compared to medulla and the pattern of expression is different between the two compartments. In the cortex, some IL-7 producing cells have a “basket-like” shape; in medulla the IL-7 producing cells are more dispersed and lacked the basket-like shape (magnification 200×). B. At higher magnification (1000×) it is possible to appreciate how the bodies of ECFP-positive cells are very infrequent compared to hematopoietic cells, but there are extensive reticular processes extending throughout the tissue. These reticular processes contain the ECFP protein and are in close contact with many thymocytes whose nuclei are visualized by DAPI (blue staining).

**Figure 3:**
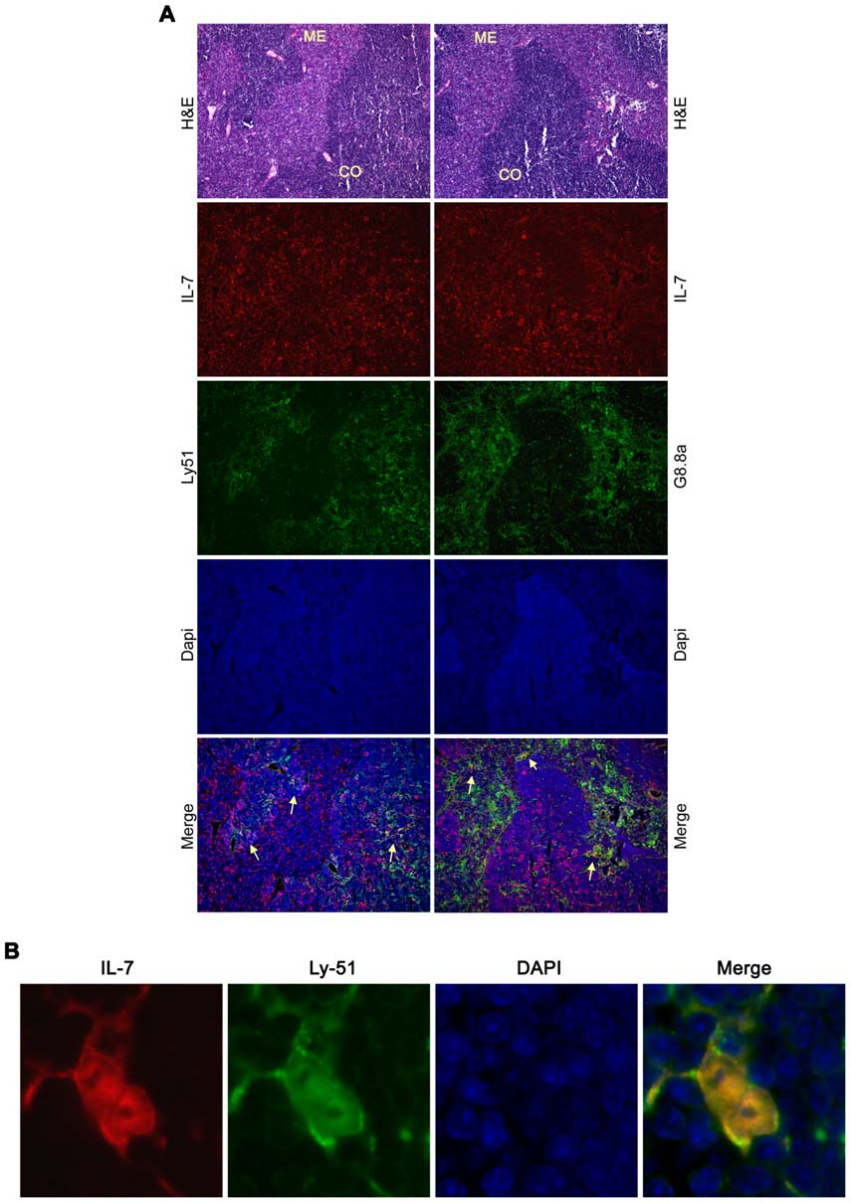
Cortical and medullary epithelial cells express IL-7 in thymus. A. Colocalization of ECFP-positive cells (red staining) with Ly51 and G8.8a (green staining) indicates the cortical cells and medullary epithelial cells, respectively, are responsible for production of IL-7 in thymus. H&E staining shows thymocyte distribution between cortex and medulla. Single color staining show the expression of ECFP (counterstained red), cortical Ly51 positive cells or medullary G8.8a positive cells (green), nuclei visualization by DAPI (blue). In the merged figures, the arrows indicate some cells with co-localization of red and green (yellow staining). Magnification 100×. B. Detail of the body of two cortical epithelial cells (magnification 400×). The yellow staining in the merged figure visualized the co-localization between ECFP (counterstained red) and the cortical marker Ly51 (green).

**Figure 4:**
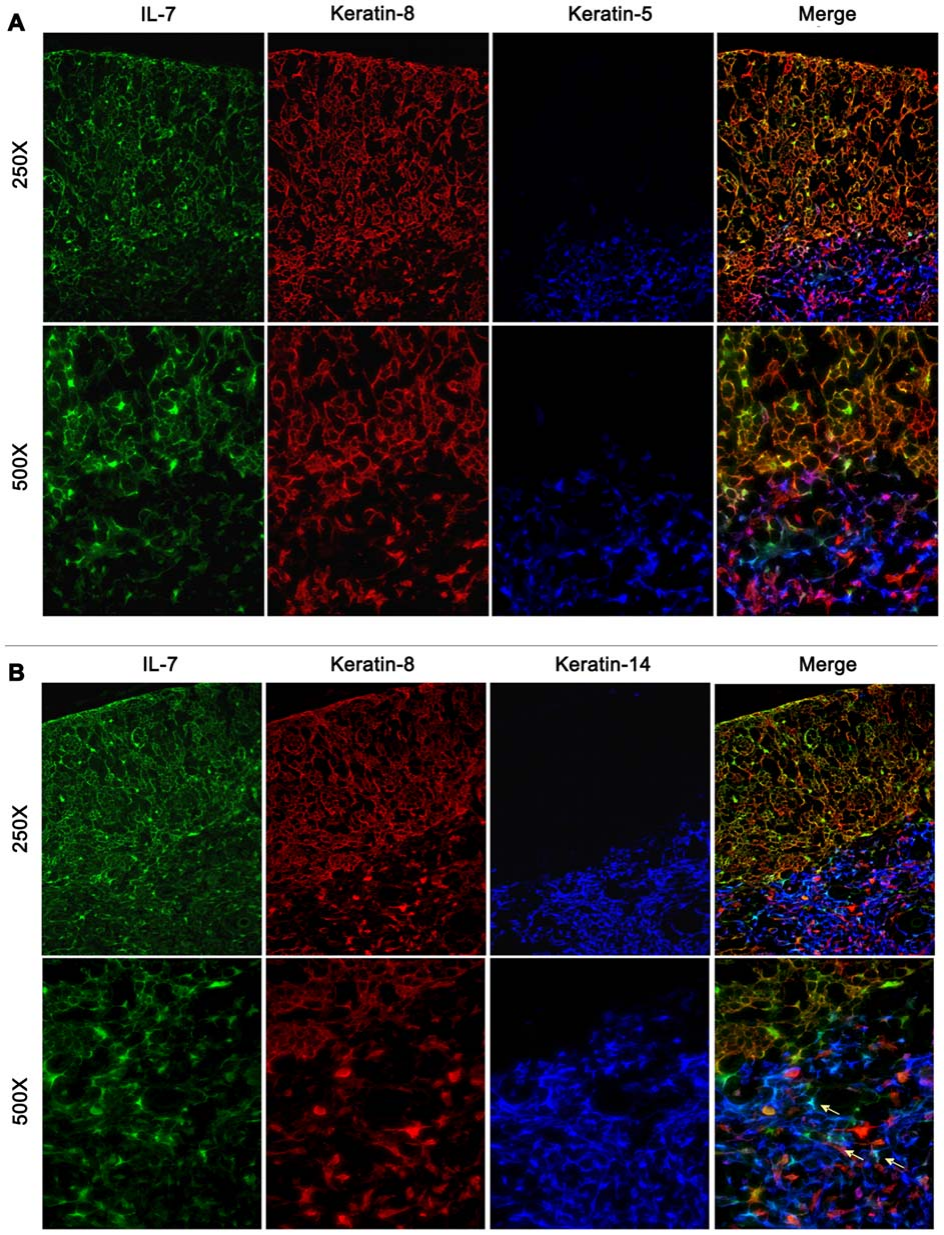
Cortical and medullary cell expression of IL-7 in thymic epithelial cells is confirmed by staining with different anti-keratin antibodies. A. ECFP (counterstained green) extensively co-localized with keratin-8 (red), a prominent cortical cell marker. To a less extent, co-localization is also present between ECFP (green) and keratin-5 (blue), a marker expressed to a much higher level on medullary epithelial cells than on cortical cells. Magnification 250× (left column) and 500× (right column) are shown. B. Co-localization between ECFP (green) and keratin-14 (blue), a prominent marker for medullary cells is shown in two different set of figures at different magnification (250×, left column and 500×, right column). Arrows in the merged figure highlight IL-7 producing cells positive for the keratin-14 medullary marker.

**Figure 5:**
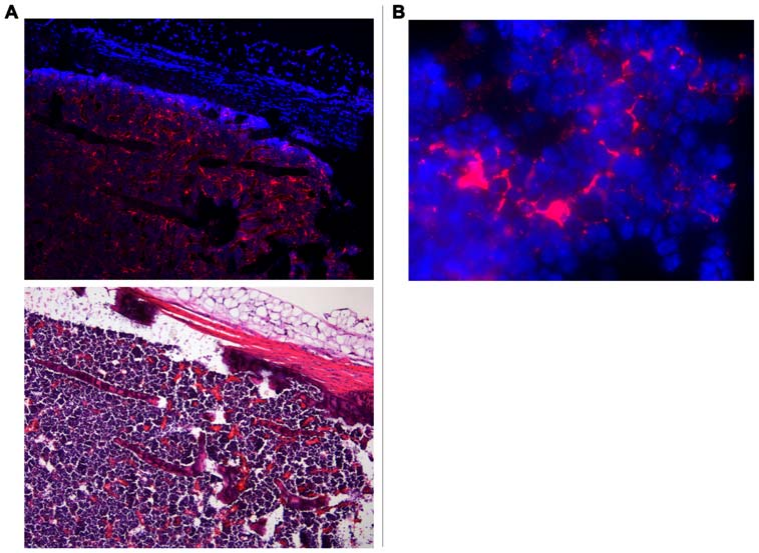
Bone marrow contains IL-7-producing cells. A. A section of femur shows that ECFP (counterstained red) is produced in bone marrow by stromal cells concentrated around vessels. The lower panel shows H&E staining of the same section. Magnification 100×. B. As also seen in thymus, bone marrow IL-7 producing cells expressed long dendrites that make extensive contacts with hematopoietic cells. Magnification 1000×.

**Figure 6:**
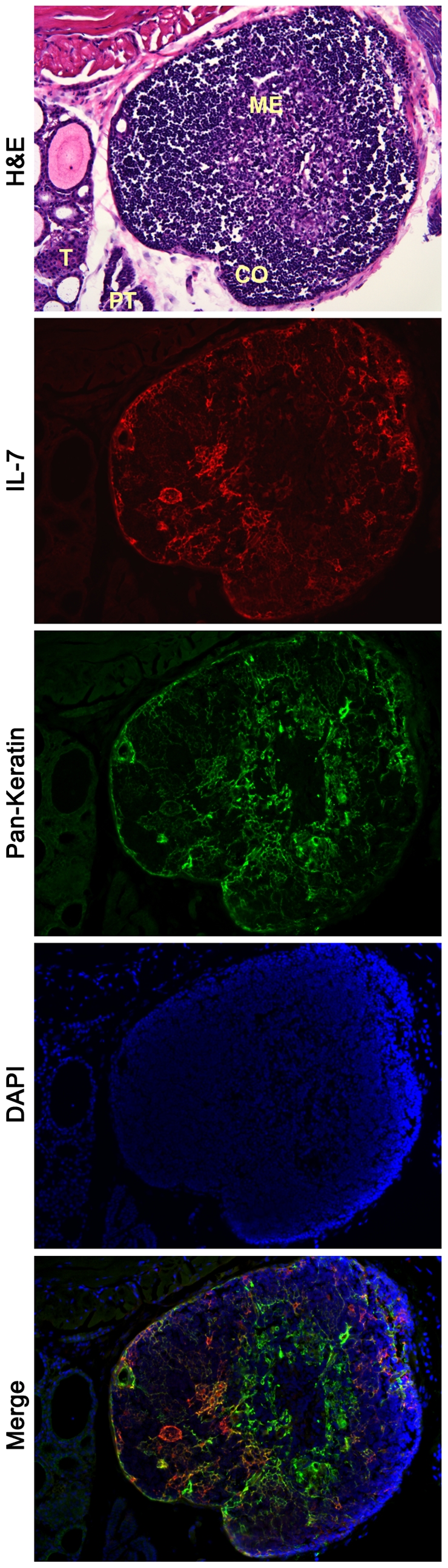
Cervical thymus contains IL-7-producing cells. H&E staining shows the cortical (CO) and medullary (ME) organization of the cervical thymus. ECFP (counterstained red) is mainly expressed in cortex by stromal cells (anti-pan keratin Ab, green staining). The wide spectrum anti-keratin antibody stains keratin-8 and keratin-5 among others, showing stromal cells present both in cortex and medulla. Magnification 200×. T, thyroid; PT, parathyroid.

ECFP expression was detected in stromal cells in thymus and bone marrow (Figures 2, 3, 4 and 5). Moreover, cervical thymus showed readily detectable levels of ECFP expression (Figure 6). In the thoracic thymus, ECFP positive cells were present in both cortex and medulla, although the pattern of expression was different (Figure 2A). In the medulla, the cell bodies containing ECFP were relatively infrequent, however an extensive network of processes extended from the cell bodies and most thymocytes were in close contact to such a process. In the cortex, ECFP positive cells appeared to envelop thymocytes, like a basket of fruit.

ECFP-positive cells were not visible in either spleen or lymph nodes of all three BAC transgenic strains - this negative finding was verified using several fluorescence microscopy systems. We also did not observe ECFP-positive cells in any other tissues examined including gut, lung, skin and brain; platelets isolated from blood were also negative. Thus, although most peripheral T cells require IL-7 for survival, we could not observe IL-7 production in the secondary lymphoid organs. Quantitative PCR was used to compare reporter expression with that of endogenous *Il7* (data not shown). The results indicate that reporter transcripts were well expressed in thymus and bone marrow where we had visualized the reporter protein. However, the reporter was only very slightly expressed in spleen and lymph node compared to *Il7*. The basis of this discrepancy will be discussed.

### Cortical and medullar epithelial cells produce IL-7 in thymus

Thymic tissue from IL7promECFP BAC transgenic mice was stained using the antibodies specific for stromal cell subsets, previously used for cell sorting. Ly51 (CDR1) is a specific marker for cortical epithelial cells. Double staining of thymic tissue using anti-EGFP Ab and anti-Ly51 Ab revealed a co-localization of ECFP and Ly51 signal (Figure 3A, left panel and Figure 3B) identifying cortical epithelial cells as IL-7 producing cells. Staining with anti-EpCAM (G8.8a) Ab, a marker specific for both cortical and medullar epithelial cells, shows that some of the EpCAM positive cells in the medullar region are also ECFP positive, suggesting that medullar epithelial cells also produce IL-7 (Figure 3A, right panel).

The expression of IL-7 in cortical and medullar epithelial cells was confirmed by staining the thymic tissue with a panel of anti-keratin antibodies. In particular, ECFP co-localized with keratin-8 and keratin-14, two prominent cortical and medullar markers, respectively (Figure 4A and 4B). Co-localization is also present for ECFP and keratin-5, a marker mainly expressed in medulla, but also present in cortical cells (Figure 4A).

In thymus, fibroblast, endothelial cells, dendritic and myeloid-derived cells did not express ECFP since there was no co-localization with MTS-15, CD31, CD45.2 or CD11c, respectively (Figure S2).

Recently, cervical thymus was identified and characterized in mice [35], [36]. The tissue organization is quite reminiscent of the thoracic thymus and the cervical thymus has been shown to support T-cell development. Since cervical thymus plays a role in T-cell differentiation, we have analyzed the expression of IL-7 and found that IL-7 is present in this ectopic thymus. As seen for thoracic thymus, expression of IL-7 is more evident in cortex compared to medulla (Figure 6).

Bone marrow also contained ECFP-positive cells as noted above (Figure 5). The shape of these cells is reminiscent of epithelial cells in thymus, but further characterization was not possible due to the lack of specific markers for bone marrow stromal cell subsets.

### Central memory T cells interact with IL-7 producing cells in bone marrow

The fluorescence of IL-7-producing cells could permit visualization of their interaction with T cells using intravital microscopy with two photon imaging. The thymus is not amenable to intravital microscopy because in the living mouse, lies over the beating heart and it cannot be isolated from the thoracic cavity. Bone marrow, on the other hand, can be imaged in the skull of the living mouse. It has been reported that central memory T cells (TCMs) express IL-7 receptor and require it for survival [37]. Since TCMs home to bone marrow [38], we asked whether these T cells might physically associate with IL-7-producing cells. TCMs were generated in vitro, labeled with CFSE and intravenously injected into ECFP positive mice. Intravital microscopy showed that TCMs homed to bone marrow as previously described. A number of TCMs showed stable interactions with IL-7 producing cells (see supplemental Movie S1 and Movie S2 which shows circles around several interactions), and analysis indicated approximately a 2.85 preference for the IL-7-producing cells (see supplemental Table S1). This suggests that IL-7 may be recognized during close physical contact between T cells and IL-7 producing cells.

This apparent attraction of TCMs to IL-7-expressing cells could be mediated by IL-7 itself. To test this possibility, we generated mice that were deficient in IL-7 but expressed the reporter. These mice were injected with CFSE labeled TCM cells and we monitored the number of cells that were present in bone marrow 24 hour later. The results showed no decrease of TCMs that initially entered in IL-7^−/−^ compared to control mice, in fact the percentage was somewhat higher possibly because they lacked endogenous CD8 cells (Figure S3A and S3B). This suggests that IL-7 itself was not the attractant guiding TCMs to the IL-7-producing cell.

If the IL-7-producing cells also expressed a chemokine that attracted TCMs, these cells express CXCR4 and can be recruited and retained into bone marrow by CXCL12 [38]. However, injection of the CXCR4 antagonist AMD3100 into IL7-ECFP mice did block the early entry of TCM cells (Figure S3A and S3B) and immunofluorescence (data not shown). These data suggest that a ligand other than IL-7 and CXCL12 may be involved in guiding TCMs to the IL-7-producing stromal cells in bone marrow.

## Discussion

IL-7 is essential for T cell development and homeostasis; however the cells that produce IL-7 have never been directly visualized by conventional immunohistochemistry due to the low level of expression. We employed a BAC transgenic strategy for detecting cells that produced ECFP from the *Il7* locus. The ECFP reporter construct was inserted in a large BAC, over 200 kbp in length, to insure fidelity of expression by the normal tissue specific regulatory elements. The IL-7-driven ECFP lacked a signal peptide resulting in accumulation in the cytosol, thereby enabling the visualizing of IL-7 producing cells. ECFP from the BAC did not interfere with IL-7 production from the endogenous gene, and thymic development appeared normal. Cells expressing ECFP were detected in thoracic thymus, cervical thymus and bone marrow.

In thymus, cortical epithelial cells contained the highest level and showed extensive dendritic branches containing ECFP in a reticular pattern. Since most cortical thymocytes were in close contact with an IL-7-containing branch, it suggested a short range, paracrine delivery of IL-7, possibly on the surface of the producing cell or nearby extracellular matrix. Some of these cortical epithelial cells had a “basket-like” shape and appeared to envelop a number of thymocytes. Medullar epithelial cells also contained ECFP, but the amount was less than in the cortex. The cervical thymus, long noted in man, has only recently been reported in mouse [35], [36]. The architecture of the cervical thymus also features cortical and medullar regions, and like the thoracic thymus, the production of ECFP was higher in the cortex.

Central memory T cells require IL-7 (reviewed in Bradley et al. [39]) and accumulate in bone marrow [38], and we show here that they can interact with IL-7 producers. IL-7 could participate in localizing TCMs in bone marrow because it has been shown to induce T cell adhesion by activating integrins [34], [40]. The initial migration of OT-1 central memory cells to bone marrow was not reduced by IL-7 deletion (supplementary Figures S3A and S3B). Based on this experiment, we conclude that IL-7 is not chemotactic for these cells. The association of central memory cells with IL-7-producing cells is therefore more likely to be due to some other attractant that is also produced by these cells. We considered SDF-1 a candidate because central memory cells express its receptor, CXCR4, and SDF-1 is expressed in bone marrow. However an antagonist against CXCR4 did not interfere with this migration to bone marrow (supplementary Figures S3A and S3B). It therefore remains an important open question as to how central memory cells are attracted to IL-7-producing cells, and we hope that gene profiling the latter cells will offer some clues to this mechanism.

In the literature, the stromal cell types in bone marrow have not been characterized as extensively as those in thymus. One candidate for the IL-7 producing cell was the osteoblast which had been reported to produce IL-7 after culture in parathyroid hormone [41]. However, staining marrow sections from the IL7promECFP reporter mouse with antibodies against markers reported for osteoblasts (osteocalcin, osteopontin or SDF-1) failed to give specific staining as determined by an experienced immunohistochemistry service at NCI. These cells appear to be fragile, as is also the case with the thymic ECFP-expressing cells, most of which disintegrated when cell suspensions were made, despite the use of gentle enzymatic dissociation methods. In the future we hope to use laser capture to characterize these intriguing cells.

There was a lack of reporter signal in spleen, lymph node, gut, lung and skin, despite the established IL-7 requirement for mature T cells found in those sites. Naïve CD8 T cells require IL-7 and from other studies [42], [43] and our own (Li et al. [44] and others not shown), this IL-7 encounter must occur within about four days following transfer into a recipient mouse and homing into lymphoid organs; without IL-7, naïve CD8 cells undergo cell cycle arrest and die. Unfortunately, our reporter was not expressed in these tissues when compared to expression of the endogenous *Il7* gene. This lack of expression (outside thymus and bone marrow) was not due to effects of the BAC integration site in the mouse genome since three different founders showed similar expression patterns. It was also not due to the placement of the reporter within the *Il7* gene, since insertion at either the 3′ or 5′ regions showed a similar mRNA expression pattern (data not shown). It was not due to toxicity of the reporter, since the expression of the endogenous *Il7* gene was not perturbed. One possibility is that the BAC contains the regulatory elements for expression in some, but not all cell types. If a regulatory element lies outside the BAC (see map in supplementary Figure S1), it would be over 80 kb upstream or downstream of the gene–a few genes have been reported with such distant sites, and although very unusual, *Il7* may be one. Another possibility is that the reporter RNA is unstable in some cells.

Having identified the principal thymic and bone marrow cells producing IL-7, a number of questions can now be addressed, including their developmental origin, their fate in senescence, and their response to immune modulators. After IL-7 is released from these cells, it is proposed that its rate of consumption by T cells determines the size of the T cell pool (reviewed in Mazzucchelli and Durum [45]). However, to evaluate this concept of IL-7 consumption, other animal models should be developed that both detect IL-7 in peripheral lymphoid tissue, and can sufficiently amplify the signal from secreted IL-7 to permit visualization.

## Materials and Methods

### Mice

C57BL/6 mice were purchased from the Animal Production Area, NCI-FCRDC (Frederick, MD). Rag2^−/−^ were originally purchased from The Jackson Laboratory (Bar Harbor, MN, USA) and maintained by homozygous breeding at NCI-Frederick, MD. Three strains of IL-7-promoter-ECFP (IL7promECFP) mice have been produced at NCI, Frederick MD and homozygous strains have been selected and maintained at NCI-Frederick, MD.

Transgenic mice expressing an IL-7 cDNA under the control of the human keratin 14 (K14) [46] promoter were maintained in specific pathogen free (SPF) conditions at the animal facility of the Department of Dermatology, Brigham and Women's Hospital, Boston, MA. Tissues from these animals were removed and processed for histopathology or RNA extraction promptly after euthanasia and shipped overnight on dry packs or dry ice to our facilities.

OT-1xRAG^−/−^ (C57BL/6-*Tg(OT-I)-RAG1^tm1Mom^*) [47] mice were purchased from Taconic Farms (Germantown, NY, USA) and maintained by homozygous breeding at NCI-Frederick, MD.

NCI-Frederick is accredited by AAALAC International and follows the Public Health Service Policy for the Care and Use of Laboratory Animals. Animal care was provided in accordance with the procedures outlined in the Guide for Care and Use of Laboratory Animals (National Research Council; 1996; National Academy Press; Washington, D.C.).

### *Il7* in situ hybridization

In situ hybridization (ISH) assays were developed that were specific for murine *Il7* and glyceraldehyde-3-phosphate dehydrogenase (*Gapdh*) and used to define temporal and special expression patterns in optimally processed adult mouse thymus and lymph node tissue. Sequence verified riboprobe generation templates corresponding to bps 884–1238 of *Gapdh* (Accession # NM_001001303) and bps 294–705, 792–1185 and 294–1185 of *Il7* (Acc # NM_008371) were produced via a PCR based strategy that utilized forward primers flanked by the T3 promoter and reverse primers flanked by the T7 promoter. Corresponding riboprobe pairs (sense and antisense) were then synthesized (Megascript high yield in vitro transcription kit, Ambion), purified (MEGAClear purification kit, Ambion), resuspended at 100 ng/µl in RNA storage solution (Ambion) and held at −80°C until use. Probe size and integrity have subsequently been confirmed via Agilent Bioanalyzer 2100 NanoChip analyses (data not shown). Due to anticipated very low level expression of *Il7* mRNA, HEK293 human cell line transfected with a high-level *Il7* mRNA expression construct (pECFP-N1/IL-7 or pEYFP-N1/IL-7) were prepared. Untransfected cells, transfected cells and mouse thymi and lymph nodes were harvested and fixed for 24 hrs at 4°C in freshly prepared 4% paraformaldehyde (PFA) in PBS then processed into paraffin blocks. Additional fixed tissue was cryoprotected in 20% sucrose at 4°C for 24 hrs and then embedded in OCT to generate frozen tissue blocks. Ten micron paraffin or frozen sections were then placed onto Super-Frost Plus glass microscope slides (Fisher Scientific) and held at −20°C until ISH. Specificity of each probe was verified in a series of pilot ISH (method described in detail below) on paraffin sections of the transfected and untransfected HEK293 cells (data not shown). Within this, previous work had indicated that *Gapdh* mRNA is readily detected in HEK293 cells by chromogenic in situ hybridization (unpublished observation). Detection of the corresponding mRNA by ISH is therefore a useful positive control that indicates successful tissue fixation, pre-treatment and in situ hybridizations.

Immediately prior to ISH, representative sections were removed from −20°C, dried at 60°C for 1 hr, de-paraffinized and re-hydrated through graded ethanols into 1X Phosphate Buffered Saline with 0.1% Tween 20 (PBST). Tissue was then permeabilized with 10 µg/ml Proteinase K (Roche) in PBST for 10 min, washed twice in PBST for 2 min, post-fixed in fresh 4% PFA for 10 min, washed twice in PBST for 2 min, acetylated in 0.1 M triethanolamine (Sigma) containing 0.25% fresh acetic anhydride (Sigma) for 30 min, and washed three times in PBST for 10 min. For frozen section ISH, frozen tissue sections were submerged in room temperature 4% PFA PBS, pH 9.5 for 1 hr, washed twice in PBS pH 7.4 for 3 min then acetylated as above. Prior to hybridization, sections were pre-hybridized at 65°C for 1.5 hrs in 250 µl of hybridization buffer (50% distilled formamide, 5X SSC, 1% SDS, 50 µg/ml yeast tRNA, 50 µg/ml heparin sodium salt) then transferred into 250 µL of hybridization buffer containing 0.5 ng/µl of probe under Cover Well™ hybridization chambers (GraceBiolabs, Bend, OR) at 65°C for 18 hrs. Unbound probe was removed by washing like slides sorted by individual probe twice in 1X SSC for 15 min at room temperature, followed by 0.5X SSC for 60 min, at 65°C, and 0.5X SSC for 5 min at room temperature. Specific hybridization was then visualized via Digoxigenin specific IHC. For IHC, hybridized slides were equilibrated in maleic acid buffer, pH 7.5 (100 mM maleic acid, 150 mM NaCl, and 0.1% Tween 20) and non-specific antibody binding was blocked for 2 hrs in maleic acid buffer containing 1% non-fat dry milk. Equilibrated slides were then incubated in a 1∶4,000 dilution of sheep anti-digoxigenin F(ab)_2_ alkaline phosphatase antibody (Roche) in blocking buffer overnight at 4°C. Unbound antibody was removed by extensive washing in maleic acid buffer for 15 min. Sections were next equilibrated in chromogenic buffer (100 mM Sodium Chloride, 100 mM Tris (Sigma, pH 9.5), 50 mM Magnesium Chloride Hexahydrate and 0.1% Tween 20) then exposed to 250 µl of chromogenic substrate (BM Purple AP Substrate, Roche) for times ranging from 1 day to one week with a daily change of substrate. Following sufficient deposition of blue/purple signal, slides were rinsed with water, counter-stained with filtered Nuclear Fast Red Stain (0.1% NFR in 5% aluminum sulfate, Kernechtrot, Germany), dehydrated through graded alcohols, cleared in xylene, and cover slipped in Permount (Fisher Scientific). A positive result is indicated by the presence of blue/purple precipitate on a pink/red background and amount of signal generated per unit of time can be used to estimate abundance of a targeted transcript. Using this approach, highly abundant transcripts (e.g. *Gapdh* in tissue or cells or *Il7* in pECFP-N1/IL-7 and pEYFP-N1/IL-7 transfected cells) are readily detected with 1 day (or less) of chromogenic exposure. Failure to detect signal after 7 days in combination with anticipated signals within technical controls is consistent with very low or absent mRNA expression and indicated by absence of blue/purple color on a pink/red background.

### RNA extraction from whole thymus and real time RT-PCR

C57BL/6, Rag2^−/−^ and K14 mouse thymi were harvested into RNA later (Ambion) and DNA-free total RNA was isolated using Triazol reagent (Invitrogen) via a modification to the provided protocol. Briefly, following addition of chloroform and phase separation, 50X DNase I buffer (1 M Tris, pH 7.0 and 100 mM MgCl_2_) was added to each aqueous phase to 1X final. One µl of Ambion DNase I (2 U/µl) was then added and incubated at RT for 15 min. Following completion of the standard protocol, concentration and purity of RNA yield was established by spectrophotometry (NanoDrop, NanoDrop Technologies, Wilmington, DE, USA) and quality confirmed by capillary electrophoresis (Agilent Bioanalyzer 2100 NanoChip).

Two microgram aliquots of each total RNA stock were converted into cDNA via hex primed reverse transcription (Thermoscript RT kit, Invitrogen). Each reaction was diluted with TE to produce a cDNA stock with a final volume of 100 µl and aliquots of each stock were then analyzed for relative amounts of *Il7*, *Gapdh*, and *18 s* ribosomal RNA via Taqman Gene expression analyses (Applied Biosystems) using a Stratagene MX3000P thermocyler running MxPro software (ver 3.0). Ct values generated from each sample with the *18 s* specific probe set were used to normalize expression of the two target genes (*Il7* and *Gapdh*) using a ΔCt method with correction for variation in amplification efficiency. Normalized ratios were then used to determine the variance in target gene expression versus wild type, and the variance was then used to approximately calculate the corresponding ratio of each transcript to the other.

### Cell preparation from thymus and cell sorting

Single cell suspensions were prepared from thymic tissue following published procedures [48], [49]. In brief, 10 thymi from C57BL/6 mice were harvested, punched with a scalpel, transferred to a beaker and stirred in 50 ml of RPMI 1640 (Mediatech Inc., Herndon, VA, USA) at 4°C for 30 min to remove the majority of the thymocytes. The resulting fragments were then digested in a series of three incubations in 5 ml of 0.125% (w/v) collagenase D/DNase I (Roche) with 0.1% (w/v) DNase I (Roche) in RPMI-1640 followed by a single digestion in 5 ml of 0.125% collagenase/dispase (Roche) with 0.1% (w/v) DNase I in RPMI-1640. Digestions were performed at 37°C for 15 min in an oven in tubes placed on the rotisserie for gentle agitation. Cells were collected after each digestion once thymic fragments had settled, pooled and kept on ice. After washing in PBS, cells were filtered through a 100 µm mesh and counted. Before staining, cells were depleted of hematopoietic cells using MACS CD45 MicroBeads (Miltenyi Biotec, Auburn, CA, USA) and autoMACS separation columns. After enrichment, cells were labeled using different combinations of the following antibodies: APC-CD45.2 (eBioscience), PE-CD11c (clone HL3, BD Pharmingen, San Jose, CA, USA), EpCAM (clone G8.8a) followed by secondary staining with PE-Cy5-anti-Rat IgG, FITC-Ly51(clone 6C3, BD Pharmingen), MTS-15 followed by secondary staining with FITC-ant-Rat IgG, and MTS-12 (anti CD31) followed by secondary staining with PE-anti-Rat IgG. Cell sorting was performed using a MoFlow Cytometer and cells were collected in RPMI-1640, washed and resuspended in lysing solution (RNAqueous-Micro Kit, Ambion, Austin, TX, USA).

### RNA extraction from sorted stromal cells and absolute quantification of *Il7* mRNA by real time RT-PCR

RNA was extracted using RNAqueous-Micro Kit (Ambion) following manufacturer's protocol. After extraction, 100 ng of RNA were retrotranscribed using Superscript III First-Strand Synthesis System (Invitrogen, Carlsbad, CA, USA) and following manufacturer's instructions.

Specific primers for real time PCR quantification of *Il7* mRNA were designed using Primer Express software (Applied Biosystems, Forster City CA, USA) and optimized for amplification and minimal primer dimer formations. Primers and probes were designed over a conserved region of the genome and synthesized by Applied Biosystems. Accurate quantification of *Il7* mRNA was accomplished using a plasmid pcDNA3 vector containing the murine *Il7* cDNA (a gift from J. Bream, Johns Hopkins University). DNA sequencing from both the 5′ and 3′ ends verified the identity. The real time PCR assays for *Il7* plasmid DNA/cDNA were carried out in 10 µl reactions using *Il7* specific primers with TaqMan Universal master mix and run on the ABI Prism 7900 (Applied Biosystems, Forster City CA, USA) (50°C for 2 min, 95°C for 10 min followed by 45 cycles at 95°C for 15 sec, 60°C for 30 sec). The murine glyceraldehyde-3-phoshate dehydrogenase (*Gapdh*) plasmid DNA used as a standard was purchased from Serologicals (Gaithersburg, MD, USA). The real time PCR assay for *Gapdh* plasmid DNA was carried out in 10 µl reactions using the murine *Gapdh* control kit (Applied Biosystems) and run as described above. A standard curve was generated by plotting the log_10_ target dilution, of murine *Gapdh* control template and murine *Il7* on the X-axis against the cycle threshold (C_t_) value from serial dilutions (6 log dynamic range) of murine *Gapdh* and *Il7* target DNA on the Y axis. Sensitivity and linear dynamic range were checked on the serial dilutions (10–10^6^ copies/reaction) of *Il7* and *Gapdh* plasmid DNA (a standard curve equation Y = −Mx+b is applied to identify the number of molecules of *Gapdh* and *Il7* present in unknown samples). The *Il7* PCR efficiency was 0.968 with a slope of −3.2, while the *Gapdh* PCR efficiency was 0.975 and the slope was −3.3. The *Il7* mRNA expression was then normalized to the *Gapdh* expression values of the same unknown samples to quantify the absolute expression of all samples in the experiment.

### Construction of the IL7promECFP mice by BAC recombineering

A bacterial artificial chromosome (BAC) containing the mouse *Il7* gene (RP23-32I7, clone length 228391 bps) was obtained from Invitrogen. BAC DNA was purified using the Nucleobond BAC kit (Clontech) according to manufacturer's instructions and characterized by SpeI fingerprinting.

The construction of the IL7-ECFP-BAC was done using galK selection as described by Warming et al. [50]. Briefly, a *galK* cassette with homology to the first translated exon of *Il7* (exon 1) was amplified using Expand High Fidelity (Roche), pgalK as template, and the following primers (ATG starting site of *Il7* is in bold, sequences priming the pgalK plasmid are underlined):

IL7->galK F: 5′-CCT GCT GCA GTC CCA GTC ATC ATG ACT ACA CCC ACC TCC CGC AGA CC**A TG** C CTG TTG ACA ATT AAT CAT CGG CA-3′ and IL7->galK R: 5′-TCC CCG GCG CGC TAG GCG CAC CTA CTT GTG CGC ACC AGA GAG CAG CGC TTT CAG CAC TGT CCT GCT CCT T-3′. PCR conditions were 94°C 15 sec, 60°C 30 sec and 72°C 1 min for 25 cycles. The PCR reaction was treated with DpnI and gel purified. Purified PCR product was transformed into heat-shocked and electrocompetent SW102 containing RP23-32I7 and Gal^+^ colonies were selected as described. Insertion of *galK* resulted in a deletion of the remainder of exon 1 as well as deletion of the splice donor of intron 1. Next, the *galK* cassette was replaced with a PCR product containing homology arms identical to the homology arms used in the first step, amplified from pECFP-1 (Clontech) using the following primers and PCR conditions as described above: galK->ECFP F: 5′-CCT GCT GCA GTC CCA GTC ATC ATG ACT ACA CCC ACC TCC CGC AGA CC**A TG** G TGA GCA AGG GCG AGG A-3′ and galK->ECFP R: 5′-TCC CCG GCG CGC TAG GCG CAC CTA CTT GTG CGC ACC AGA GAG CAG CGC TTG CCT TAA GAT ACA TTG ATG AGT TTG GA. This PCR reaction product was DpnI-treated, gel-purified, and transformed into SW102 Gal^+^ cells and DOG resistant colonies were selected as described [50]. One out of 10 analyzed BAC clones was correctly targeted after galK counter selection, and the modified area of the BAC was confirmed by direct BAC sequencing of large-prep BAC DNA using ABI PRISM BigDye Terminators (Applied Biosystems). The IL-7-ECFP BAC construct was linearized with PiSceI (New England Biolabs) and microinjected into fertilized ova of C57BL/6 females at the pronuclear stage. Mice were screened for acquisition of the transgene by Southern Blot Analysis of DNA obtained from tail biopsies using standard procedures. Genomic DNA was digested with BglII and analyzed with a 299 bp probe amplified from the RP23-32I7 BAC. The wild type band is 2.6 kb and the transgenic band is 3.6 kb.

### Genotyping and selection of homozygous IL7promECFP transgenic mice

Genomic DNA from mouse tail biopsies was extracted by ethanol precipitation after digestion in digestion buffer (50 mM Tris-HCl, pH 8, 100 mM EDTA, 100 mM NaCl, 1% SDS) with 500 µg Proteinase K (Roche Applied Science, Indianapolis, IN, USA) overnight at 55°C and resuspended in 100 µl of 0.1X SSC buffer. Approximately 10 ng of this DNA was analyzed in a 20 µl SYBR green PCR reaction containing 1X Brilliant SYBR Green QPCR Master Mix (Stratagene), 30 nM ROX passive reference dye, and 300 nM each primer. The ECFP transgene was assayed using specific primers, and differences in input DNA were normalized using an endogenous reference gene, *Folh1*, in separate wells. Primer sequences used were CFP-F 5′-ATG CCA CCT ACG GCA AGC TG-3′, CFP-R 5′-TTC TGC TGG TAG TGG TCG GCG-3′, FolH1-F 5′-CCA AGC AGC CAC AAC AAG TA-3′ and FolH1-R 5′-TCC ATA GGG ATT TTG TGA TTC TG-3′. Real time PCR was performed in triplicate on a MX3000P Stratagene (Cedar Creek, TX, USA) instrument running an initial enzyme activating step of 95°C for 10 min, followed by 45 cycles of denaturation at 95°C for 30 sec, annealing at 60°C for 30 sec, and extension at 72°C for 30 sec. Normalized data was then analyzed using the −2^ΔΔCt^ method to determine relative fold change compared against a known hemizygous animal (calibrator).

### Immunohistochemistry and immunohistology

All tissue samples were fixed in 4% PFA for approximately 16 hrs at 4°C, then transferred to 20% sucrose and incubated for another 16 hrs at 4°C. Finally, tissues were blotted to remove excess sucrose, frozen into OCT compound (Sakura, 4583), and held at −80°C until sectioning. Seven µm sections were cut using a cryostat and stored at −80°C until the day of staining.

For IL-7 immunohistochemistry, sections were warmed to room temperature for approximately 60 min, then rinsed in 1X PBS for 3 changes, 3 min each. Antigen retrieval was performed using a citrate based buffer pH 6 (Biogenex, HK086-9K) in Milestone RHS-1 microwave processor for 10 min to reach 100°C, followed by 10 min at 100°C. Peroxide block was applied at 0.6% in 0.1% saponin (Sigma, S4521)/1X PBS (wash buffer) at room temperature for 15 min followed by rinsing in wash buffer 3 times, 3 min each. Anti-mouse IL-7 antibodies (rabbit polyclonal IgG from Santa Cruz, goat polyclonal IgG from Santa Cruz and R&D, monoclonal rat IgG_2b_ from R&D, biotinylated rabbit polyclonal IgG from PeproTech, monoclonal mouse IgG from Amgen) were applied at different dilutions, in wash buffer, at room temperature for at least 60 min. Samples were then rinsed using wash buffer 3 times, 3 min each. Avidin-Biotin Complex reagent was applied (ABC, Vector) following manufacturer's instructions diluted in wash buffer, and incubated at room temperature for 30 min, followed by another rinse for 3 times, 3 min each with 1X PBS. Slides were then developed in DAB (Sigma, D5905-100TAB), counterstained, dehydrated, and cover slipped.

For ECFP-target cell identification and co-localization, tissue sections were warmed to room temperature for approximately 60 min, placed in acetone for 10 min at room temperature, and then dried at room temperature for approximately 5 min. Sections were re-hydrated in 1X PBS for 10 min followed by application of 2% normal goat serum/PBS (Vector, S-1000) for 20 min at room temperature. Serum in excess was removed (not rinsed) and primary antibodies were applied for 60 min at 37°C (unless differently specified) as a cocktail diluted in 0.1% BSA/1X PBS. For examining ECFP expression, anti-GFP (Abcam) was used at a 1∶1000 dilution. Other antibodies were used at the following dilutions: FITC-anti-Ly51 (BD Pharmingen) at 1∶1000; anti-G8.8a (gift from Gray D.) at 1∶1000; anti-MTS-15 (gift from Gray D.) 1∶20; biotinylated-CD45.2 (eBioscience) at 1∶500; FITC-CD11c at 1∶1000; CD31 (Santa Cruz) at 1∶200 O/N at 4°C. The sections were then rinsed in 1X PBS for 10 min and corresponding secondary antibodies were applied as a cocktail, diluted 1∶300 in 1% BSA/1X PBS, for 30 min at room temperature. The following secondary antibodies have been used: donkey anti-rabbit 488 (Molecular Probes), goat anti-rabbit 546, and biotinylated goat anti-rabbit (Vector) followed by streptavidin conjugated Alexa 546 (Molecular Probes) for anti-GFP; goat anti-rabbit 488 (Molecular Probes) for G8.8a; goat anti-rat 546 (Molecular Probes) for MTS-15; streptavidin conjugated Alexa 546 (Molecular Probes) for CD45.2; donkey anti-goat 546 (Molecular Probes) for CD31. Slides were rinsed in 1X PBS for 10 min, wiped dry, and cover slipped with Prolong Gold (Molecular Probes).

All samples were visualized with a Nikon Eclipse 80i microscope under consistent illumination and exposure conditions for each respective stain. Brightfield images were captured using Nikon DXM1200F digital camera and Nikon ACT-1 software. Fluorescent images were obtained using Exfo X-Cite 120 excitation, Nikon UV-2E/C, B-2E/C and G-2E/C filter cubes, Qimaging Retiga 2000R digital camera, and Media Cybernetics Image-Pro plus v5.1 software.

For anti-keratin staining, intact thymic lobes were fixed by immersion in 4% PFA, cryoprotected with phosphate buffered saline containing 30% (w/v) sucrose. Samples were then embedded in OCT (Sakura Finetek U.S.A., Inc., Torrance, CA, USA), frozen, and then sectioned at 5–7 µm with a cryostat. Sections were collected on SuperfrostPlus slides (Fisher Scientific, Pittsburg, PA, USA). After drying overnight, sections were hydrated in PBS and then incubated in a mixture of primary antibodies. To Troma1 hybridoma supernatant (Developmental Studies Hybridoma Bank, University of Iowa; dshb.biology.uiowa.edu/), was added goat anti-green fluorescent protein (Rockland, Gilbertsville, PA, USA), and different rabbit antibodies, either anti-keratin 5, anti-keratin-8 or anti-keratin 14 (all from Covance, Berkley, CA, USA). Controls consisted of mixtures of normal goat, rabbit, and rat IgGs diluted to equivalent concentration. Primary antibodies were applied for 1 hr at room temperature. After repeated washes in PBS, slides were incubated with a mixture of conjugated secondary antibodies diluted in PBS containing 10 mg/ml bovine serum albumin and 10% (v/v) normal donkey serum. Secondary antibodies were donkey anti-goat IgG Alexa 488, donkey anti-rabbit IgG Alexa 555, and chicken anti rat IgG Alexa 647 (all from Invitrogen-Molecular Probes, Eugene, OR, USA). Following incubation for 1 hr. protected from light, the slides were repeatedly washed with PBS, then incubated in 10 mM acetate buffer, pH 6, containing 1 mM CuSO_4_ for 10 min before a final wash in PBS. Coverslips were mounted with Fluoromount G (SouthernBiotech, Birmingham, AL, USA). Sections were viewed with a Leica microscope equipped with a digital camera (Orca-ER, Hamamatsu Photonic Systems, Bridgewater, NJ, USA) to collect images. Resulting monochrome images were converted to RGB images with Photoshop (Adobe, San Jose CA, USA).

### Intravital multiphoton microscopy

Lymphocytes were isolated from OT-IxRAG^−/−^ mice and differentiated into central memory T-cells (TCMs) by stimulation with OVA peptide (SIINFEKL, a generous gift T.Mitchell, U.Louisville) followed by culture in the presence of IL-15 for 5 to 7 days [38]. Differentiation of cells into TCMs was evaluated by FACS analysis for CD8, CD44, CD62L, CD122 and CCR7 expression. Intravital microscopy of mouse bone marrow was performed using a protocol modified from a previous report [38]. Twenty-four hrs after i.v. injection of CFSE-labeled TCMs, mice were anesthetized with isoflurane (Baxter, 2.5% vaporized in an 80∶20 mixture of O_2_ and air), and the hair in the neck and scalp was removed with hair removal lotion (Nair, Carter Products, NY). The frontoparietal skull was exposed and the mouse head was immobilized in a custom stereotactic holder. The imaging system was an LSM510 NLO Meta (Carl Zeiss, Jena, Germany) driven by a Chameleon femtosecond pulsed laser (Coherent Inc., Santa Clara, CA) tuned to 880 nm, and an inverted microscope (Axiovert 200; Carl Zeiss) equipped with a 40X water immersion objective (Achroplan IR, NA 0.8; Carl Zeiss). The microscope was enclosed in an environmental chamber in which anesthetized mice were warmed by heated air. Fluorescent cells were detected using a bandpass emission filter at 480/40 nm (for ECFP) or 525/50 nm (for CFSE). Vessels were visualized after i.v. injection of 70 kDa Texas Red conjugated-dextran (620/60 nm filter). Image stacks were collected with a 3 µm vertical step size at a depth of 100–150 µm below the skull bone surface. For 3D videos, 4 sequential image stacks were acquired at 3 mm z spacing to cover a volume of 154 µm×154 µm×9.0 µm with a 1 min interval between repetitive image stack collections. Imaging data were processed with Imaris (Bitplane, Zurich, Switzerland).

## Supporting Information

Figure S1 Map of BAC used to generate transgenic mice. This BAC was selected because the Il7 gene was flanked by large spans that were likely to contain the regulatory elements.
(0.10 MB TIF)

Figure S2 No IL-7 expression in non-epithelial cells in thymus. IL-7 is not expressed by myeloid-derived cells, dendritic cells, fibroblasts or endothelial cells in thymus since no co-localization of ECFP (red) with CD45.2, CD11c, MTS-15 or CD31, respectively (green) was observed. DAPI (blue) identifies cell nuclei. Magnification 100X.
(6.02 MB TIF)

Figure S3 OT-1-TCM migration to bone marrow: Lack of effect of CXCR4 block or Il7 deletion. Central memory cells were generated by in vitro culture of OT-1 cells as described. Cells were labeled with CFSE and injected into wild type mice that were previously injected with HBSS (as a control) or with the CXCR4 antagonist AMD3100 and compared with IL-7−/− recipients. Twenty-four hours later, bone marrow was harvested from the long bones, stained with anti-CD8 and analyzed by flow cytometry. A. Three individual recipients are shown for each treatment. No inhibition of migration of OT-1-TCM cells resulted from blocking CXCR4 or deleting Il7. Note that the IL-7−/− recipient lacked endogenous CD8 cells. B. The data is shown in numerical form representing the total number of OT-1-TCM cells recovered per individual mouse.
(1.14 MB TIF)

Table S1 OT-1-TCM association with ECFP producers. Data are corrected by removing OT-1 whose center overlies a stromal cell (probable spill-over artifact).
(0.03 MB DOC)

Movie S1 Central memory cells (TCMs) were obtained by in vitro differentiation of T cells from OT-1xRAG−/− mice. TCMs were labeled with CFSE and injected intravenously twice (−24 hr and −2 hr) into IL7promECFP mice. Bone marrow was visualized by intravital microscopy. CFSE labeled TCMs (green) exit the blood stream (red) and some interactions with ECFP producing cells (blue) were observed. The video spans a 40 minute time.
(3.42 MB MPG)

Movie S2 Central memory cells (TCMs) were obtained by in vitro differentiation of T cells from OT-1xRAG−/− mice. TCMs were labeled with CFSE and injected intravenously twice (−24 hr and −2 hr) into IL7promECFP mice. Bone marrow was visualized by intravital microscopy. CFSE labeled TCMs (green) exit the blood stream (red) and some interactions with ECFP producing cells (blue) were observed. The video spans a 40 minute time.
(2.82 MB AVI)
