# Supplementary figures and images for: Visualization and Identification of IL-7 Producing Cells in Reporter Mice
Source: PLoS One. 2009 Nov 10;4(11):e7637. doi: 10.1371/journal.pone.0007637 (PMC2770321; doi:10.1371/journal.pone.0007637)

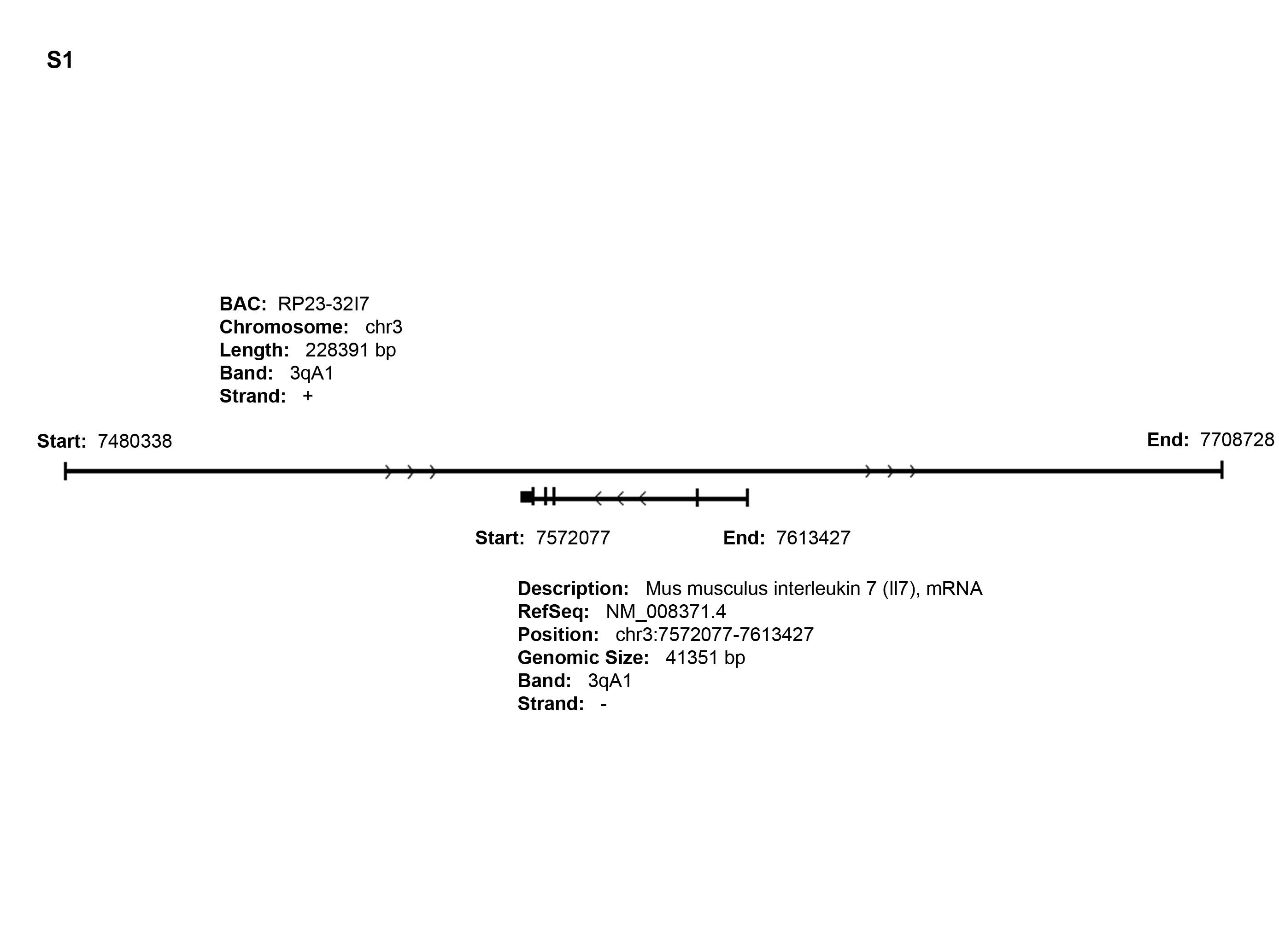

Supplement: Figure S1 — Map of BAC used to generate transgenic mice. This BAC was selected because the Il7 gene was flanked by large spans that were likely to contain the regulatory elements. (0.10 MB TIF) [file pone.0007637.s001.tif]

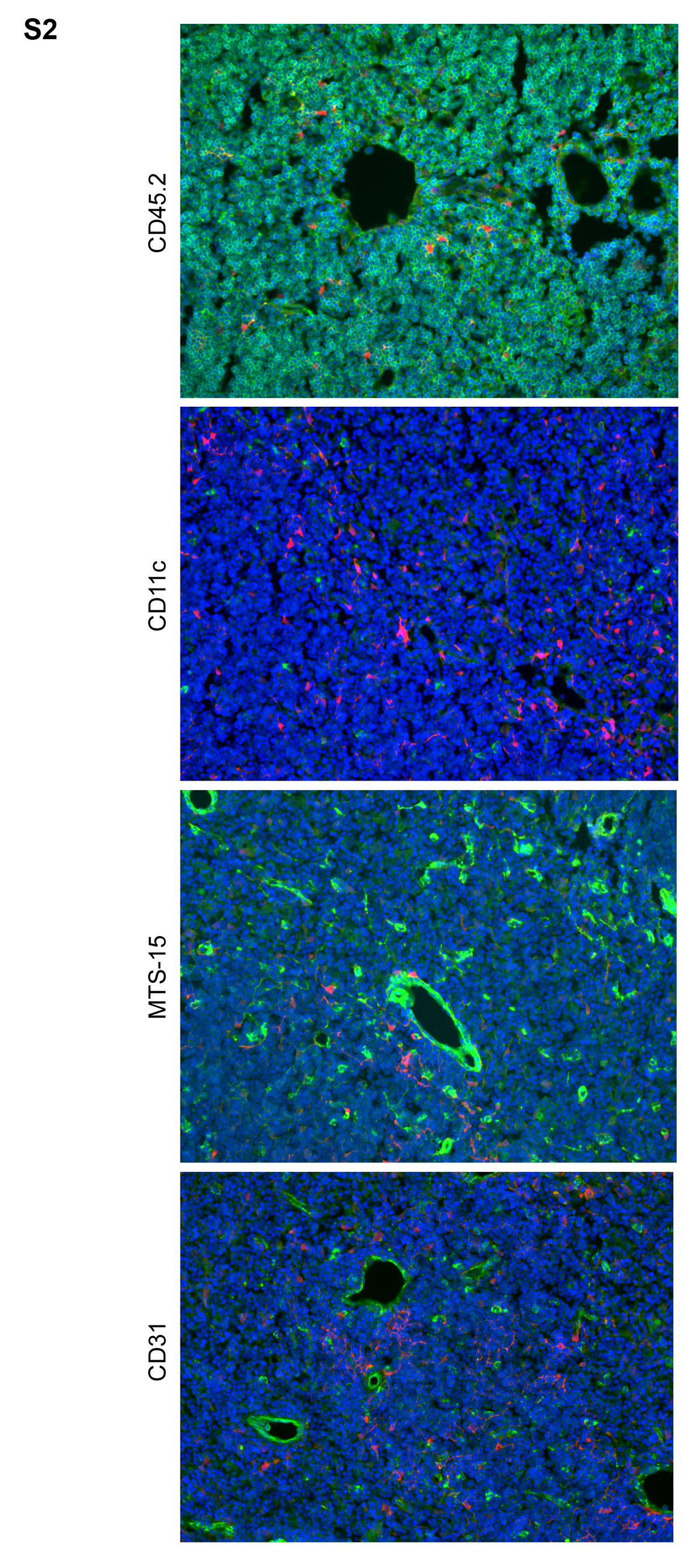

Supplement: Figure S2 — No IL-7 expression in non-epithelial cells in thymus. IL-7 is not expressed by myeloid-derived cells, dendritic cells, fibroblasts or endothelial cells in thymus since no co-localization of ECFP (red) with CD45.2, CD11c, MTS-15 or CD31, respectively (green) was observed. DAPI (blue) identifies cell nuclei. Magnification 100X. (6.02 MB TIF) [file pone.0007637.s002.tif]

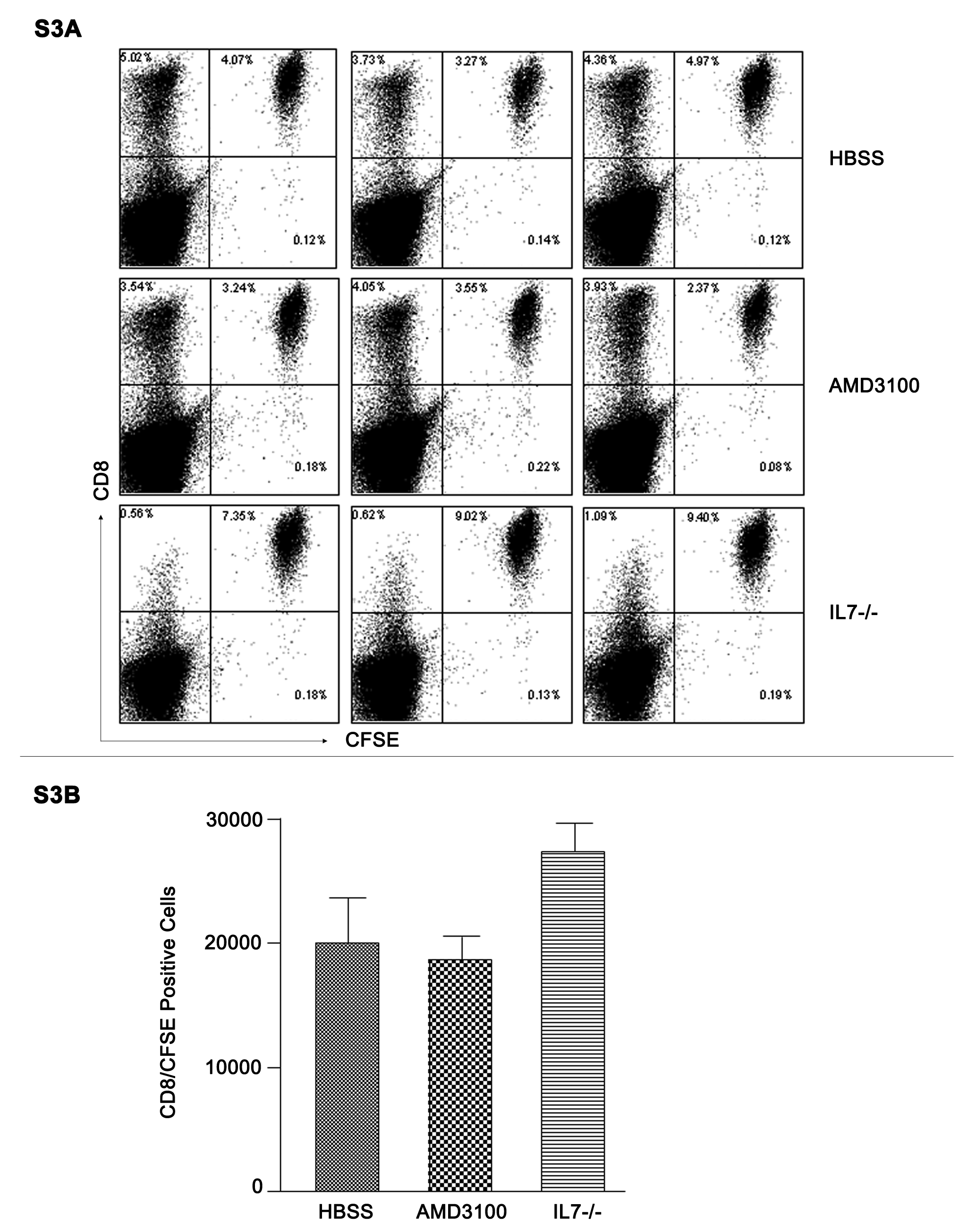

Supplement: Figure S3 — OT-1-TCM migration to bone marrow: Lack of effect of CXCR4 block or Il7 deletion. Central memory cells were generated by in vitro culture of OT-1 cells as described. Cells were labeled with CFSE and injected into wild type mice that were previously injected with HBSS (as a control) or with the CXCR4 antagonist AMD3100 and compared with IL-7−/− recipients. Twenty-four hours later, bone marrow was harvested from the long bones, stained with anti-CD8 and analyzed by flow cytometry. A. Three individual recipients are shown for each treatment. No inhibition of migration of OT-1-TCM cells resulted from blocking CXCR4 or deleting Il7. Note that the IL-7−/− recipient lacked endogenous CD8 cells. B. The data is shown in numerical form representing the total number of OT-1-TCM cells recovered per individual mouse. (1.14 MB TIF) [file pone.0007637.s003.tif]
