# Supplementary material for: Visualization and Identification of IL-7 Producing Cells in Reporter Mice
Source: PLoS One. 2009 Nov 10;4(11):e7637. doi: 10.1371/journal.pone.0007637 (PMC2770321; doi:10.1371/journal.pone.0007637)
Supplement: Table S1 — OT-1-TCM association with ECFP producers. Data are corrected by removing OT-1 whose center overlies a stromal cell (probable spill-over artifact). (0.03 MB DOC) [file pone.0007637.s004.doc]

**Table S1**

| **Data set** | **Total** | **Proximal** | **Distal** | **Proximal** | **Distal** | **Prox/Dist** |
| --- | --- | --- | --- | --- | --- | --- |
| 1 | 13 | 4 | 9 | 4.72 | 5.08 | 0.93 |
| 2 | 8 | 4 | 4 | 5.24 | 2.15 | 2.44 |
| 3 | 6 | 2 | 4 | 3.63 | 1.93 | 1.88 |
| 4 | 10 | 8 | 2 | 7.08 | 1.33 | 5.28 |
| 5 | 8 | 5 | 3 | 6.17 | 1.66 | 3.73 |
| **Mean** | 9 | 4.6 | 4.4 | 5.37 | 2.43 | 2.85 |
| **Std Dev** | 2.65 | 2.19 | 2.70 | 1 | 2 | 1.69 |

OT-1 density (OT-1 Number x10^5^)

**Table S1.** **OT-1-TCM association with ECFP producers.** Data are corrected by removing OT-1 whose center overlies a stromal cell (probable spill-over artifact).
